# Using narrative medicine to enhance self-compassion and reflective capacity in pediatric residents

**DOI:** 10.1093/pch/pxag083

**Published:** 2026-08-20

**Authors:** Madeleine Bondy, Foluke Akinboyo, Elif Bilgic, Joanna Humphreys

**Affiliations:** Department of Pediatrics, Quinte Health, Belleville, Ontario, Canada; McMaster Resident Affairs, McMaster University, Hamilton, Ontario, Canada; Department of Pediatrics, McMaster University, Hamilton, Ontario, Canada; McMaster Health Education Research, Innovation & Theory (MERIT) Centre, McMaster University, Hamilton, Ontario, Canada; Department of Pediatrics, McMaster University, Hamilton, Ontario, Canada

**Keywords:** Narrative medicine, Medical education, Self-compassion, Reflective practice, Resident wellness

## Abstract

Paediatric residency is a formative yet demanding period characterized by emotionally charged patient encounters, high expectations, and personal and professional growth. These challenges can contribute to emotional fatigue and disconnection. Strengthening physicians’ capacities for reflection and self-compassion may promote resilience and sustain compassionate care.

Self-compassion involves responding to one's own suffering with mindfulness, kindness, and awareness of shared humanity, while reflective capacity refers to the ability to critically examine professional experiences. Narrative medicine has shown promise in fostering compassion, perspective-taking, and professional fulfillment, but evidence within paediatric residency remains limited.

This study evaluated whether participation in a narrative medicine reflective group using the Parallel Chart exercise improved self-compassion and reflective capacity among paediatric residents and assessed the acceptability of this intervention.

A single-group exploratory pre-post study was conducted among McMaster University paediatric residents (postgraduate years 1 to 4). Participants attended up to six one-hour sessions co-facilitated by a paediatric resident trained in narrative medicine and a postgraduate counsellor. Sessions included reflective writing about meaningful patient encounters followed by guided discussion. Self-compassion and reflective capacity were measured using validated questionnaires, and qualitative feedback was analyzed thematically.

Participation in the Paediatric Awareness and Understanding of Salient Encounters (PAUSE) narrative medicine sessions improved self-compassion and reflective capacity scores. Qualitative analysis identified themes of enhanced mindfulness, compassion, shared humanity, and meaning in clinical work. Participants described the sessions as restorative, validating, and supportive.

The PAUSE narrative medicine intervention suggests that structured narrative medicine may provide a sustainable approach to promoting wellness, reflection, and humanism in paediatric residency training.

## Introduction

Paediatric residency represents a formative yet demanding stage of medical training, during which residents encounter emotionally intense patient care experiences, high expectations, and the continual pressure to develop both professional competence and personal resilience. These demands can lead to emotional fatigue, self-doubt, diminished empathy, and a gradual loss of connection to meaning in one's work. Despite the clear importance of emotional processing in sustaining professional well-being, trainees often have limited structured opportunities to explore the psychological impact of their clinical experiences, an issue widely acknowledged in medical education (1,2).

Self-compassion and reflective capacity have been identified as important protective factors that support wellness, emotional regulation, and professional identity formation. Self-compassion, described by Neff (2016) as responding to one's own suffering with mindfulness, kindness, and an awareness of shared humanity, has been linked to increased resilience and reduced psychological distress among health care professionals (3,4). Reflective capacity, the ability to critically examine one's clinical experiences, has long been recognized as central to professional growth, supporting ethical decision-making, continuous learning, and sustained meaning in clinical practice (5–7).

Narrative medicine has emerged as a promising pedagogical model for fostering these qualities. First articulated by Charon (2001), narrative medicine integrates storytelling, close reading, and reflective writing to help clinicians engage with both patient experiences and their own internal responses (8). By creating structured space for meaning-making, narrative medicine has been shown to strengthen empathy, perspective-taking, and awareness of shared humanity. In paediatrics, narrative medicine has been proposed to reconnect residents with the emotional dimensions of caregiving and to counteract the detachment that can occur in high-stress training environments (9).

A small but growing body of research supports the use of narrative medicine in residency programs. Studies across internal medicine, surgery, obstetrics and gynecology, and emergency medicine show improvements in empathy, reflection, and professional fulfillment following structured narrative interventions (10–13). In paediatrics, early work suggests narrative medicine may enhance mindfulness, compassion, and well-being (14,15). However, systematic reviews highlight persistent gaps, including a lack of rigorous evaluation of reflective-capacity outcomes and underuse of specific narrative tools, such as the Parallel Chart, in structured interventions (16). Most of the literature has focused on reading poetry and literature (16).

The Parallel Chart, a reflective writing practice distinct from the medical record, invites clinicians to explore emotional, relational, and moral dimensions of patient encounters in an unfiltered and private format. Despite its potential for emotional depth, the Parallel Chart remains underutilized in paediatric training programs.

This exploratory single-group pre- and post-study addresses these gaps by evaluating the PAUSE (Paediatric Awareness and Understanding of Salient Encounters) narrative medicine program, which centers on the Parallel Chart. The objectives were to assess whether participation improved self-compassion and reflective capacity among paediatric residents and to evaluate acceptability of the model within postgraduate medical education.

## Methods

This study employed an exploratory single-group pre-post design to examine the potential impact of a narrative medicine intervention on paediatric residents' self-compassion and reflective capacity. A pre-post approach was selected to capture both quantitative changes in participant-reported outcomes and qualitative descriptions of residents' experiences with the intervention. Given the exploratory nature of the study and the absence of a comparison group, the study was designed primarily to assess implementation within an existing residency curriculum and to generate preliminary evidence regarding potential educational and emotional benefits.

The intervention took place at McMaster Children's Hospital, a tertiary academic paediatric center in Canada. The general paediatrics residency program includes trainees from Postgraduate Years (PGY) 1 through 4. Sessions were scheduled immediately before the weekly Academic Half Day in a dedicated teaching space to provide protected time for participation and minimize disruption to clinical responsibilities.

Approximately 50 paediatric residents were eligible to participate. Recruitment occurred through email invitations distributed by the residency program and verbal reminders during educational sessions. Participation in the PAUSE curriculum was voluntary, and completion of research surveys was not required for participation in the educational intervention itself. Twenty residents attended at least one session, representing all four years of training. Because residents were permitted to attend any number of sessions, exposure to the intervention varied across participants.

The PAUSE sessions were delivered once per academic block (approximately every four weeks) for a total of six sessions. Each 60-minute session followed a consistent structure designed to promote psychological safety, emotional reflection, and intentional meaning-making. Sessions began with a brief grounding or mindfulness exercise to help residents transition from clinical work into a reflective mindset. Residents then completed a ten-minute Parallel Chart writing exercise focused on a meaningful patient encounter. They were also offered the choice of art materials and modelling clay to engage with if they preferred not to write. Prompts encouraged reflection on emotions, uncertainty, ethical tensions, moments of connection, or other aspects of clinical experiences that remained personally salient. Residents were informed that Parallel Chart entries were private, confidential, and entirely separate from the medical record.

Following the writing exercise, residents participated in a facilitated group discussion lasting approximately 35 to 40 minutes. Participation in discussion was flexible; residents could choose to share excerpts from their writing, reflect verbally, listen quietly, or simply remain present. Discussions emphasized confidentiality, nonjudgment, and respect for differing reflective styles. Optional creative modalities, including modelling clay, were also available for residents who preferred tactile or nonverbal forms of reflection.

Each session was co-facilitated by a paediatric resident with narrative medicine training and a counsellor with expertise in learner wellness and emotional support. This co-facilitation model was intended to balance peer relatability with psychological support and emotional containment. Facilitators met regularly throughout the study period to maintain consistency in session structure and reflective approach.

Quantitative data were collected at baseline and following completion of the six-session series. Self-compassion was measured using the Self-Compassion Scale–Short Form (SCS-SF), a 12-item instrument assessing mindfulness, self-kindness, and common humanity. Reflective capacity was assessed using the Reflective Practice Questionnaire (RPQ) reflective capacity subscale, which evaluates dimensions including reflection-in-action, reflection-on-action, self-appraisal, and reflective communication (3,5). Both instruments have demonstrated validity and reliability in health professions trainees (3,5).

Participants completed surveys only twice: prior to their first PAUSE session and following the final session of the intervention period. No interim assessments were conducted. Paired *t*-tests were used to examine pre–post changes in self-compassion and reflective capacity among participating residents, with each participant serving as their own control. Cohen's *d* was calculated to estimate effect sizes.

Because attendance varied across participants, linear regression analyses were conducted secondarily to explore associations between the number of sessions attended and magnitude of pre–post change in each outcome measure. Attendance variability was intentionally permitted to reflect the realities of residency scheduling and to explore potential dose–response relationships under real-world educational conditions.

Qualitative data were collected through anonymous written responses to open-ended post-intervention survey questions exploring residents' emotional experiences, perceived benefits, impressions of the co-facilitation model, and suggestions for improvement. Responses underwent reflexive thematic analysis using Braun and Clarke's six-phase framework (17). Data were first familiarized through repeated reading by two members of the research team. Initial codes were generated inductively in NVivo and applied independently to the full dataset. Coding focused on participants' descriptions of emotional experience, perceived impact, and meaning derived from the sessions.

To enhance trustworthiness, the research team engaged in reflexive discussion regarding researcher positionality and maintained an audit trail documenting analytic decisions. Credibility was further supported through investigator triangulation and inclusion of representative participant quotations. Although responses were relatively brief, strong thematic convergence supported analytic sufficiency.

Ethics approval was obtained from the Hamilton Integrated Research Ethics Board (#17260). Given the emotionally sensitive nature of reflective work, facilitators monitored participants for distress and provided information regarding available wellness supports following each session. Survey responses were anonymized, and participation had no impact on academic standing.

## Results

### Quantitative Outcomes

As summarized in Table 1, residents demonstrated strong engagement, attending an average of 4.3 sessions (SD = 1.2). This high level of attendance supports embedding narrative medicine within existing educational structures.

**Table 1:** Participant characteristics and session attendance (*N* = 20).

Total participants	20 (100%)
PGY level	PGY1: 6 (30%)
	PGY2: 5 (25%)
	PGY3: 5 (25%)
	PGY4: 4 (20%)
Mean age (years)	28.4 ± 1.6
Sessions attended (mean)	4.3 ± 1.2
Attended ≥4 sessions	15 (75%)
Attended 1 to 2 sessions	3 (15%)
Attended all 6 sessions	6 (30%)

Abbreviation: PGY, postgraduate years.

Significant improvements were observed in both quantitative outcomes (Table 2). Mean self-compassion scores increased from 22.50 (SD = 3.40) to 33.40 (SD = 4.04), a 10.9-point rise that was statistically significant, *t*(19) = 16.81, *P* = 7.3 × 10^−13^, with a very large effect size (*d* = 3.76). Reflective capacity increased from 27.60 (SD = 5.25) to 41.30 (SD = 5.97), yielding a 13.7-point improvement, *t*(19) = 17.92, *P* = 2.3 × 10^−13^, with an effect size of *d* = 4.00.

**Table 2:** Pre–post changes in self-compassion and reflective capacity.

Measure	Pre mean ± SD	Post mean ± SD	Mean difference	T(19)	*P*-value
Self-compassion (SCS-SF total)	22.50 ± 3.40	33.40 ± 4.04	+10.9	16.81	7.3 × 10^−13^
Reflective capacity (RPQ total)	27.60 ± 5.25	41.30 ± 5.97	+13.7	17.92	2.3 × 10^−13^

These changes are visually illustrated in Figure 1, which shows clear upward shifts for both outcomes at the individual and group levels. Nearly every resident demonstrated improvement, regardless of baseline levels.

**Figure 1:**
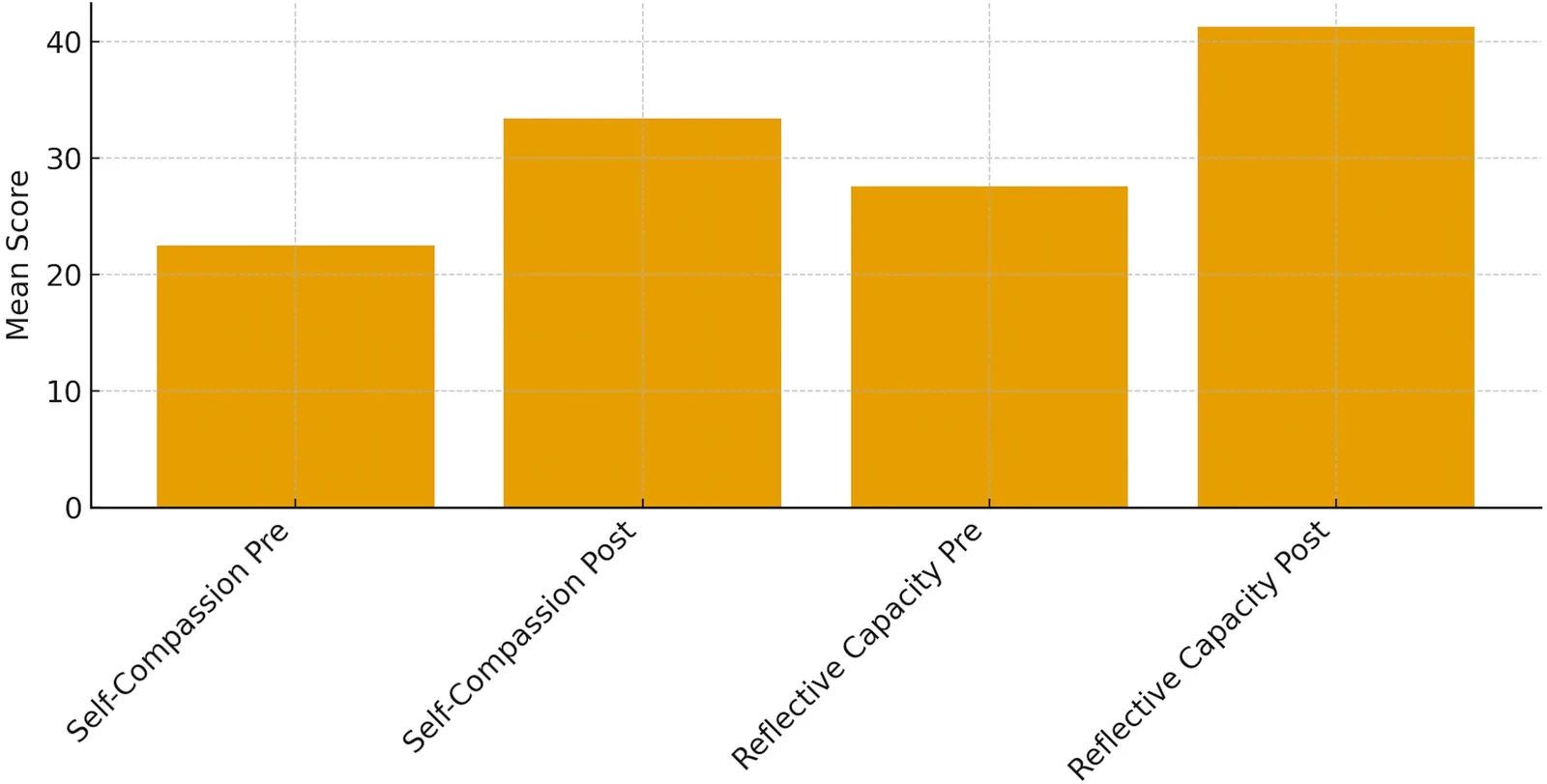
Mean self-compassion and reflective capacity scores before and after participation in a six-session narrative medicine program. Mean scores on the Self-Compassion Scale–Short Form (SCS-SF) and Reflective Practice Questionnaire (RPQ) increased following participation in the program.

A dose–response relationship emerged: residents who attended more sessions exhibited significantly larger pre–post improvements in both self-compassion (*β* = 0.41, *P* = .02) and reflective capacity (*β* = 0.47, *P* = .01). This suggests that repeated, structured exposure to reflective practice enhanced the intervention's effectiveness.

Collectively, the quantitative results indicate that the PAUSE program produced substantial and clinically meaningful improvements in both outcomes. The magnitude and consistency of these gains support the effectiveness of Parallel Chart–based narrative medicine within paediatric training.

### Qualitative Results

Qualitative analysis revealed four interrelated themes that described residents' experiences of the PAUSE sessions: emotional resonance and depth; connection and shared humanity; cultivating reflective capacity and self-compassion; and value of co-facilitation and structure.

#### Emotional resonance and depth

Residents described being unexpectedly moved by the reflective writing process. The Parallel Chart elicited emotional honesty and depth. One resident shared, “I was surprised by the emotions it invoked in me.” Several described the sessions as calming or grounding, noting that the reflective pause provided relief within the demands of clinical service: “The reflective work added a sense of calm to my day.”

Creative modalities, including modelling clay, offered alternate avenues for emotional exploration. As one resident put it, “I didn’t expect how reflective the modeling clay could be.” The sessions helped surface emotions, grief, uncertainty, gratitude, that often go unacknowledged in daily practice.

#### Connection and shared humanity

Residents emphasized the sense of community created through shared storytelling. Hearing colleagues express similar feelings of uncertainty or emotional struggle reduced isolation. One participant described, “the sense of relief from listening to others,” while another wrote, “How helpful it is to not feel alone on this journey.”

The group was repeatedly described as a “safe space.” One resident wrote, “It felt safe and non-judgmental, a space to talk about challenging experiences with my coresident.” The validation and normalization of shared vulnerability strengthened relational bonds and reinforced the central narrative medicine principle of shared humanity.

#### Cultivating reflective capacity and self-compassion

Residents reported meaningful shifts in their internal dialogues and emotional awareness. Many articulated developing greater self-kindness, such as “Give yourself kindness,” and “Mistakes are human.” These reflections suggest the sessions helped soften harsh self-evaluations and support resilience.

Residents also described deeper engagement with clinical encounters: “I’m taking away curiosity and a drive to reflect more about my patient interactions.” Others noted a sense of emotional grounding: “I’m taking away peace.” These insights reflect a broadening reflective capacity and renewed sense of meaning in clinical work.

#### Value of co-facilitation and session structure

Residents consistently praised the co-facilitation model. One participant wrote, “Loved it, it felt like a safe, collaborative space,” while another highlighted, “It was lovely to have another non-medical perspective.” The presence of both a peer and a counsellor supported emotional exploration and psychological safety.

Flexibility in participation, writing, listening, speaking, or engaging creatively, made the sessions accessible and inclusive: “It was welcoming—you could choose how to participate.”

### Suggestions for Improvement

Residents primarily requested more frequent or longer sessions, reflecting strong perceived value. Some desired more discussion time; others preferred shorter writing intervals. All feedback emphasized the program's relevance and importance.

## Discussion

This multi-methods study demonstrates that a narrative medicine intervention centered on the Parallel Chart is both feasible and beneficial within paediatric residency training. The significant quantitative improvements in self-compassion and reflective capacity highlight the potential of structured reflective practice to support emotional resilience, identity formation, and compassionate clinical care. These findings align with research identifying self-compassion as a protective factor against emotional distress and a key component of well-being among healthcare professionals (18,19). They also reinforce longstanding evidence that reflective practice is important to learning, meaning-making, and professional development (5,6).

The large effect sizes observed in both outcome measures are consistent with emerging literature suggesting that narrative medicine can strengthen resilience, empathy, and mindfulness among pediatric trainees. For example, Bajaj et al. (2023) demonstrated sustained improvements in resident well-being following a narrative medicine program, while earlier pediatric-focused work has shown benefits such as increased emotional awareness and reduced sense of isolation (15,16). Our findings build on this work by demonstrating robust improvements specifically in reflective capacity, an outcome identified as understudied in a major narrative medicine review (16).

The qualitative findings provide important insight into how these improvements occur. Residents described meaningful emotional processing, validation of shared struggles, and renewed connection to the humanistic dimensions of care. These themes echo broader arguments in the paediatric humanities literature, which contend that narrative competence is essential for fostering empathy, reducing emotional exhaustion, and sustaining meaningful practice (9). The Parallel Chart, in particular, appeared to support deeper reflective exploration by providing an emotionally safe, private, and non-evaluative space, consistent with Charon's (2001) original description of its purpose, adding to current literature (8).

The co-facilitation model emerged as an additional strength. Similar to findings from other wellness and narrative interventions, residents emphasized the importance of skilled facilitation, psychological safety, and the complementary perspectives offered by both a clinician and a counsellor (13,20). This interdisciplinary structure may be particularly valuable in paediatrics, where emotionally loaded patient encounters are common and residents may lack traditional debriefing opportunities.

Our study also adds to the literature by highlighting the potential dose–response relationship in narrative interventions. Residents who attended more sessions demonstrated greater improvements in both self-compassion and reflective capacity, suggesting that reflective capacity may strengthen over time through repeated engagement, an idea supported by conceptual domains of reflective practice as well as emerging work on wellness curricula and longitudinal humanities programming (2,6).

Taken together, these findings suggest that integrating structured narrative medicine practices, particularly those using the Parallel Chart, may offer a scalable and impactful approach for supporting paediatric residents' emotional development. By promoting self-kindness, fostering shared humanity, and deepening reflective insight, narrative medicine may help residents sustain compassion and meaning in their work, aligning with broader calls to enhance humanistic training in medicine (1,9).

This study should be interpreted in the context of several limitations. Participation in the PAUSE curriculum was voluntary, introducing the potential for self-selection bias, as residents who chose to participate may have been more interested in reflective practice, wellness programming, or narrative medicine than nonparticipants. Approximately 50 paediatric residents were eligible to participate, while 20 attended at least one session; therefore, findings may not reflect the perspectives or experiences of the broader residency cohort. Although the curriculum was successfully implemented within an existing educational structure and was valued by participating residents, feasibility was assessed in a limited manner. The study did not formally examine barriers to participation, opportunity costs, administrative burden, competing educational demands, or reasons residents declined participation. The absence of a control group and reliance on self-reported measures further limit causal inference, as improvements in self-compassion and reflective capacity may reflect participant motivation, expectancy effects, or other unmeasured factors. In addition, engagement with the Parallel Chart writing process itself was not formally assessed, and the study was conducted at a single institution with a relatively small sample size and short follow-up period, limiting generalizability and assessment of long-term sustainability. Accordingly, these findings should be interpreted as exploratory and hypothesis-generating, supporting the potential value of narrative medicine among participating residents while warranting further controlled evaluation in larger and more diverse residency settings. The absence of a control group, reliance on self-reported measures, and short follow-up period limit conclusions regarding durability of outcomes. Variable session attendance and voluntary participation may introduce self-selection bias, and findings may not generalize beyond a single institution. These limitations are appropriate to the study's exploratory aims and inform directions for future controlled evaluation.

## Conclusion

The PAUSE narrative medicine program enhanced self-compassion, reflective capacity, and emotional connectedness among paediatric residents. Residents described the sessions as meaningful, restorative, and transformative to their personal and professional growth. Given its strong acceptability and measurable impact, narrative medicine, particularly through the Parallel Chart, represents a promising, scalable approach to enriching paediatric residency education and fostering reflective, humane, and resilient physicians.
